# Supplementary material for: Healthcare providers’ (HCPs) perspectives in initiating discussion on mammogram screening, and their perceived barriers and enablers to screening in women—A qualitative study
Source: PLoS One. 2025 Jan 9;20(1):e0311999. doi: 10.1371/journal.pone.0311999 (PMC11717275; doi:10.1371/journal.pone.0311999)
Supplement: S1 File — This is an interview guide with broad questions was used to guide the discussion. (PDF) [file pone.0311999.s001.pdf]

S1 File: Interview guide

1. What do you think are the factors that encourage patients to take up screening mammograms?
2. What do you feel is your best approach/ methods to advise patients on screening mammograms?
3. What are the barriers (for themselves and for patients) that you experience when advising patients on screening mammograms?
4. What do you think can be done to overcome the barriers experienced?
5. Are you confident of providing information and initiating discussions with patients regarding mammogram screening?
6. What are the concerns of your patients when deciding on screening mammograms?
7. Are you up to date with current national guidelines on breast cancer screening?
8. Who should provide information and initiate discussions with patients on screening mammograms (primary care like yourself or specialist clinics)?
9. Do you think screening mammography is useful for detecting breast cancer?
10. Are you aware of the risks and limitations of screening mammography?
11. How many patients do you provide information and initiate discussions regarding screening mammograms in a week?
12. What is the general response that you get from your patients when you attempt to discuss screening mammograms with them?
