# Supplementary material for: Healthcare providers’ (HCPs) perspectives in initiating discussion on mammogram screening, and their perceived barriers and enablers to screening in women—A qualitative study
Source: PLoS One. 2025 Jan 9;20(1):e0311999. doi: 10.1371/journal.pone.0311999 (PMC11717275; doi:10.1371/journal.pone.0311999)
Supplement: Attachment — Submitted filename: PLOS ONE review2.docx [file pone.0311999.s004.docx]

PLOS ONE review

This study employed qualitative methods to explore healthcare professionals' views on the barriers and facilitators to mammogram breast screening uptake in Singapore. Doctors and nurses participated in focus groups. The findings highlighted several factors influencing screening uptake, such as language barriers, women's educational backgrounds, and cost/accessibility.

While the study addresses a significant topic, several improvements are needed:

**Typos**: The document contains several typos that require correction.

**Introduction**: Provide contextual information about Singapore's healthcare system, as PLOS ONE's global audience may be unfamiliar with it.

**Literature Update:** Data collection stopped in 2020. The literature review should be updated to include more recent studies.

**Methods:** Further explanation of the theoretical framework is necessary. How widely used is this framework, and why was it chosen over others?

**Results**: Detail the number of participants in each of the five focus groups. Indicate whether there was a mix of doctors and nurses, as group dynamics could influence the findings.

**Discussion**: The strengths and limitations section should include some strengths.
